# Survey data on energy-saving policies, energy price, crisis and household energy-saving behavior

**DOI:** 10.1016/j.dib.2023.109646

**Published:** 2023-10-05

**Authors:** Tung Thanh Nguyen, Dat Ngoc Nguyen, Huong Thi Lan Pham

**Affiliations:** a University of Engineering & Technology, Vietnam National University, Hanoi, Vietnam; b Foreign Trade University, Hanoi, Vietnam; c QAglobal, Hanoi, Vietnam

**Keywords:** Energy-saving, Energy policy, Energy price, Crisis, Energy-saving behavior

## Abstract

The state policies and energy prices are evaluated to play a crucial role in the context of crises occurring in each country. The authors collected data on state policies and energy prices concerning energy-saving behavior during crises, with a particular focus on the Covid-19 crisis. The data was gathered from 1216 respondents, who are households. The data's reliability was assessed using Smart-PLS software. The data will provide research ideas related to state policies, energy prices, and energy-saving behavior associated with crises similar to Covid-19.

Specifications Table

**Table:** 

Subject	Social sciences (general)
Specific subject area	Sustainability development, energy
Data format	Raw, analysed
Type of data	Table
Data collection	The survey is collected from 10/2021 to 05/2022 via direct survey and resulted in 1216 responses.
Data source location	Region: Asia Country: Vietnam
Data accessibility	The dataset is provided as a supplementary file. Direct URT to data: https://data.mendeley.com/datasets/hn7nskn2mj/1 Data identification number DOI: 10.17632/hn7nskn2mj.1

## 1 Value of the Data

• The data will provide information about the role of energy-saving policies in relation to energy consumption issues in households.
• The data will provide information about the role of energy prices concerning energy consumption issues in households.
• The data will provide information about the role of crises concerning energy consumption issues in households.
• The data will assist researchers in studying the impact of energy-saving policies, energy prices, and crises on energy-saving behavior.
• The data will help the government formulate energy-saving policies and pricing strategies to promote energy-saving behavior in the context of crises.

## 2 Objective

In the context of crises and the increasingly complex global warming phenomenon, this study gathers survey data to assess the impact of energy-saving policies and energy prices on the energy-saving behavior of households.

## 3 Data Description

Energy plays a crucial role in various aspects of daily life, leading to increasing demand and consumption levels worldwide due to economic development and population growth [1]. In Vietnam, significant progress has been made in the energy sector alongside overall advancements. With rapid economic growth, energy consumption in Vietnamese households has risen sharply, representing 30.8 % of total consumption, following industry (32.2 %) and construction (31.7 %). However, the reliance on fossil fuel-based thermal power (58.4 % of total energy production) has resulted in environmental challenges, posing a threat to sustainable development [2]. To promote energy efficiency and conservation, the Government of Vietnam has introduced Decision 280/QD-Ttg, aiming to achieve energy savings of 5 to 7 % and reduce average power consumption from 3 % to 15 % in specific areas by 2030. Energy-saving policies play a crucial role in reducing energy consumption and optimizing the use of existing energy sources [1]. It is an important aspect of policies to consider in protecting the environment, enhancing energy security, and supporting sustainable development [3].

Energy prices significantly influence household energy consumption behavior, carrying significant implications for energy conservation policies and strategies. Higher energy prices drive the adoption and usage of energy-saving appliances, leading to decreased energy consumption and cost reduction [4]. Consumers may also reduce appliance usage duration and practice turning off lights when not needed, further promoting energy conservation [5]. These behavioral changes collectively result in reduced overall energy consumption, fostering a culture of energy conservation within the community and contributing to a more sustainable society [5,6]. Understanding the relationship between energy prices and household energy use is essential for developing effective initiatives aimed at promoting energy efficiency and conservation [7].

Indeed, in recent years, researchers have also explored the impact of crises on the income and energy-saving behavior of the population [8]. Particularly, the COVID-19 factor has been extensively considered from 2020 up to the present (Considering COVID-19 as a crisis). Besides posing health risks, COVID-19 has increased the risk of job loss and reduced income during uncontrollable periods [9]. Therefore, according to the protective motivation theory, employment risks may prompt individuals to engage in economically protective behavior by using energy-saving and efficient measures.

The population was examined households in provinces and cities in Vietnam. Our data were collected from October 2021 to May 2022 via social network (facebook, Email). This study surveyed 1216 valid samples. The results show that females account for 51.6 % with 628 individuals, while males represent 48.4 % with 588 individuals. Regarding education, the largest proportion is university graduates with 755 individuals (62.1 %), followed by postgraduates with 271 individuals (22.3 %). The third-largest group is college graduates with 99 individuals (8.1 %), and the smallest group is high school graduates with 91 individuals (7.5 %). In terms of occupation, the majority are official staff with 586 individuals (48.2 %), followed by self-employed individuals with 362 (29.8 %). The smallest group is unemployed individuals with 12 (1 %). As for monthly income, the main group surveyed has an income above 20 million VND/month, accounting for 529 individuals (43.5 %), followed by the group with incomes between 15 to 20 million VND/month with 439 individuals (36.1 %). The group with incomes below 10 million VND/month has the smallest percentage with 56 individuals (4.6 %). The participant characteristics are presented in Table 1.

**Table 1:** Respondents’ profiles (n = 1216)

	n = 1216	%
*Gender*		
Female	628	51.6
Male	588	48.4
*Education*		
High School	91	7.5
Colleage	99	8.1
Graduate University	755	62.1
Master/Phd.	271	22.3
*Occupation*		
Offical staff	586	48.2
Unemployment	12	1
Factory workers	49	4
Engineer	105	8.6
Self-employed	362	29.8
Lecturer/teacher	40	3.3
Others	62	5.1
*Income*		
under 10 million VND/month	56	4.6
10- under 15 million VND/month	192	15.8
15- under 20 million VND/month	439	36.1
> 20 million VND/month	529	43.5

Data is considered important when providing information on factors related to energy-saving policies, price, crisis, subject norm, perceived usefulness, perceived ease of use, behavior control, attitude toward energy-saving, and intention for energy-saving behavior. The data can yield assessments of households regarding the current state of government energy-saving policies as well as other factors related to intentions and energy-saving behaviors in households. Additionally, the data will provide insights into the evaluation model of the impact of energy-saving policies on intentions and energy-saving behaviors in households.

## 4 Experimental Design, Materials and Methods

The items measured constructs in the model were adapted from previous studies [6,7,10,11]. All of constructs are first-order constructs. The items in the questionnaire were translated from English to Vietnamese and used back-translation to ensure the questions do not change meaning in the translation process. The questionnaire is referenced from previous research studies and presented in Table 2. The items will use the Likert scale with five levels. Where 1= totally disagree, 2 = disagree, 3 = normal, 4 = agree, 5 = totally agree. The scales and references for the design of the scale are detailed in Table 2.

**Table 2:** Reality analysis results

Construct/items		Loading
**Subject Norm, adapted from Wang et al.**[11]**; Cronbach's Alpha=0.804; CR=0.885; AVE=0.719**		
SNO1	Households need to be conscious of energy-saving behavior	0.808
SNO2	Your electricity-saving behavior is influenced by family, friends, or neighbors	0.853
SNO3	If everyone around you participates in saving electricity, you will participate more actively in saving electricity.	0.881
**COVID-19, adapted from Vo-Thanh et al. (2021); Cronbach's Alpha** **=** **0.769; CR** **=** **0.8678; AVE** **=** **0.685**		
COVID1	You worried about your income due to COVID-19	0.835
COVID2	COVID-19 affect your job	0.779
COVID3	In general, you are greatly affected by COVID-19	0.866
**Policy, adapted from Zhang et al.**[6]**; Cronbach's Alpha** **=** **0.812; CR** **=** **0.914; AVE** **=** **0.842**		
PO1	Policies to encourage economical and efficient use of energy are practical	0.924
PO2	Energy saving policies bring many benefits to people	0.912
**Perceived easy of use, adapted from Ru et al.**[13]**; Cronbach's Alpha** **=** **0.674; CR** **=** **0.859; AVE** **=** **0.752**		
PEU1	Easy-to-use energy-saving devices	0.837
PEU2	Easy repair/maintenance of energy-saving equipment	0.897
**Price adapted from Fu et al.**[7]**; Cronbach's Alpha** **=** **0.92; CR** **=** **0.949; AVE** **=** **0.862**		
PRI1	The current price of energy is high compared to the average income	0.924
PRI2	The price of energy (electricity, gas) increases every year	0.941
PRI3	You feel worried when energy prices increase.	0.92
**Perceived of usefulness adapted from Ru et al.**[13]**; Cronbach's Alpha = 0.729; CR = 0.847; AVE = 0.649**		
PU1	Using appliances with energy-saving technology will help save on monthly electricity bills.	0.775
PU2	Using appliances with energy-saving technology will help protect the environment	0.847
**Behavior control adapted from Fu et al.**[7]**; Cronbach's Alpha = 0.76; CR = 0.862; AVE = 0.675**		
CON1	You have the knowledge and skills to implement energy saving in your daily life.	0.834
CON2	Energy saving actions are easy for you.	0.822
CON3	Saving money is an important factor for you to implement energy-saving behaviors	0.809
**Attitude adapted from Ru et al.**[13]**; Cronbach's Alpha = 0.708; CR = 0.835; AVE = 0.63**		
ATT1	You think that saving energy in daily life will be helpful for environmental protection.	0.871
ATT2	You think that saving energy in daily life will help reduce greenhouse gas emissions.	0.827
ATT3	You think that saving energy in daily life is valuable to alleviate the current energy shortage problems.	0.67
**Intention adapted from Zhang et al.**[6]**; Cronbach's Alpha = 0.783; CR = 0.874; AVE = 0.697**		
INT1	You will participate in daily energy -saving activities	0.807
INT2	Willing to use skills to reduce energy consumption (turn off equipment when not in use)	0.846
INT3	Willing to pay to invest in electricity-saving products	0.852
**Behavior adapted from Zhang et al.**[6]**; Cronbach's Alpha = 0.757; CR = 0.846; AVE = 0.58**		
BE1	You turn off the device to reduce energy consumption when not in use	0.748
BE2	You used energy -saving appliances in your home.	0.813
BE3	You often remind others to use energy saving and efficiency	0.682
BE4	In general, you always take action to save energy for your family and the people around	0.796

Collected data will be evaluated for reliability with two criteria: Cronbach's Alpha coefficient greater than 0.6 and Composite Reliability greater than 0.7 [12]. Analysis results on Smart-PLS 3.0 software show that all constructs are reliable (see Table 2). Next, the constructs continue to be included in the convergence analysis through two criteria: factor loading factor greater than 0.5 and Average Variance Extracted (AVE) greater than 50 %. The analysis results also show the construct reaching convergence validity (see Table 2).

The formulas to calculate the factor loading, CR, AVE:

$$AVE\xi_{j}=\frac{1}{K_{j}}\sum_{k=1}^{K_{j}}\lambda_{jk}^{2}$$

$$CR=\frac{{\left(\sum_{i}^{p}\lambda_{i}\right)}^{2}}{{\left(\sum_{i}^{p}\lambda_{i}\right)}^{2}+\sum_{i}^{p}\sigma_{i}}$$

λ_jk_ is the factor loading; K_j_ is the number of indicators of contruct $\xi_{j}$; p is number of indicators; $\sigma_{i}$ is variance of the error term for the indicators.

Constructs are further tested for discriminant validity, as suggested by Fornell and Larcker. The test results show that the factors reach discriminant values (the square root of the AVE values is higher than most coefficients correlation with ranging between 0.764 and 0.918). We adhered to the guidance provided by Hair et al. [12] and proceeded to investigate the discriminant validity by calculating the Heterotrait-Monotrait (HTMT) ratios. These ratios were less than 0.9, thus confirming that our constructs exhibited discriminant validity, as outlined in the work of Hair et al. [12] (the detail in Table 3).

**Table 3:** The discriminant validity test

	Mean (sd)	ATT	BE	CON	COVID	INT	PEU	PO	PRI	PU	SNO
ATT	3.67(0.97)	**0.794**									
BE	3.80(0.99)	0.548	**0.761**								
		*0.726*									
CON	3.86(0.94)	0.643	0.576	**0.822**							
		*0.857*	*0.747*								
COVID	3.96(0.93)	0.581	0.592	0.63	**0.828**						
		*0.751*	*0.758*								
INT	3.89(0.91)	0.584	0.667	0.601	0.642	**0.835**					
		*0.763*	*0.858*	*0.778*	*0.825*						
PEU	3.60(1.02)	0.435	0.507	0.447	0.473	0.504	**0.867**				
		*0.615*	*0.719*	*0.616*	*0.643*	*0.686*					
PO	3.77(0.98)	0.525	0.596	0.521	0.527	0.553	0.515	**0.918**			
		*0.681*	*0.755*	*0.662*	*0.665*	*0.692*	*0.697*				
PRI	3.97(0.77)	0.494	0.73	0.511	0.513	0.629	0.426	0.49	**0.928**		
		*0.596*	*0.872*	*0.611*	*0.609*	*0.741*	*0.538*	*0.567*			
PU	3.85(0.93)	0.502	0.685	0.583	0.631	0.675	0.496	0.584	0.58	**0.806**	
		*0.679*	*0.915*	*0.781*	*0.842*	*0.892*	*0.705*	*0.758*	*0.708*		
SNO	3.94(0.99)	0.598	0.64	0.655	0.712	0.706	0.509	0.595	0.562	0.617	**0.848**
		*0.763*	*0.809*	*0.837*	*0.902*	*0.888*	*0.677*	*0.735*	*0.654*	*0.806*	

*Notes*: 1^st^ value = Correlation between variables (2-tailed *t*-test); 2^nd^ value (italic) = HTMT ratio; Square root of AVE (bold diagonal).

The HTMT with constructs $\xi_{i}$ and $\xi_{j}$, K_i_ and K_j_ indicators was calculated:

$$HTMT_{ij}=\frac{1}{K_{i}K_{j}}\sum_{g=1}^{K_{i}}\sum_{h=1}^{K_{j}}r_{ig,jh}÷\sqrt{\left(\frac{2}{K_{i}\left(K_{i}-1\right)}\times \sum_{g=1}^{K_{i}-1}\sum_{h=g+1}^{K_{i}}r_{ig,ih}\times \frac{2}{K_{j}\left(K_{j}-1\right)}\times \sum_{g=1}^{K_{j}-1}\sum_{h=g+1}^{K_{j}}r_{ig,jh}\right)}$$

r_ig,jh_ is correlation coefficient between the construct scores of constructs ξi and ξj

## Ethics Statement

Respondents to questionnaire participated voluntarily. Also, all personal information such as name, identity is not collected to ensure the respondent's privacy.

This study was promoted by the Degree No 052022/QD-QAglobal from Quantitative Analysis Center, QAglobal, Vietnam

## CRediT authorship contribution statement

**Tung Thanh Nguyen:** Conceptualization, Methodology, Writing – original draft, Visualization, Investigation. **Dat Ngoc Nguyen:** Conceptualization, Methodology, Writing – original draft, Visualization, Investigation. **Huong Thi Lan Pham:** Writing – original draft.

## Data Availability

Survey data on energy-saving policies, energy price, crisis and household energy-saving behavior (Original data) (Mendeley Data)

## Appendix Supplementary Materials

Image, application 1
