# Supplementary material for: Survey data on energy-saving policies, energy price, crisis and household energy-saving behavior
Source: Data Brief. 2023 Oct 5;51:109646. doi: 10.1016/j.dib.2023.109646 (PMC10587727; doi:10.1016/j.dib.2023.109646)
Supplement: Supplementary file 1 [file mmc1.docx]

**QUESTIONNAIRE**

**Survey data on energy-saving behavior in buildings**

**Part 1. Please tick your level of agreement with the statements stated in the questions below. in which the:**

1- Strongly disagree, 2- Disagree, 3- Normal, 4- Agree; 5- Strongly agree

| **Code** | | **Content** | | **Level** | | | | | | | |  |
| --- | --- | --- | --- | --- | --- | --- | --- | --- | --- | --- | --- | --- |
| ***I. Subject Norm*** | | | | | | | | | | | |  |
| SNO1 | Households need to be conscious of energy-saving behavior | | | 1 | 2 | | 3 | | 4 | | 5 |  |
| SNO2 | Your electricity-saving behavior is influenced by family, friends, or neighbors | | | 1 | 2 | | 3 | | 4 | | 5 |  |
| SNO3 | If everyone around you participates in saving electricity, you will participate more actively in saving electricity. | | | 1 | 2 | | 3 | | 4 | | 5 |  |
| ***II. COVID19*** | | | | | | | | | | | |  |
| COVID1 | | You worried about your income due to COVID-19 | | 1 | | 2 | | 3 | | 4 | 5 |  |
| COVID2 | | COVID-19 affect your job | | 1 | | 2 | | 3 | | 4 | 5 |  |
| COVID3 | | In general, you are greatly affected by COVID-19 | | 1 | | 2 | | 3 | | 4 | 5 |  |
| ***III.* Policy** | | | | | | | | | | | |  |
| PO1 | Policies to encourage economical and efficient use of energy are practical | | | 1 | 2 | | 3 | | 4 | | 5 |  |
| PO2 | Energy saving policies bring many benefits to people | | | 1 | 2 | | 3 | | 4 | | 5 |  |
| ***IV. Perceived easy of use*** | | | | | | | | | | | |  |
| PEU1 | Easy-to-use energy-saving devices | | | 1 | 2 | | 3 | | 4 | | 5 |  |
| PEU2 | Easy repair/maintenance of energy-saving equipment | | | 1 | 2 | | 3 | | 4 | | 5 |  |
| ***VII. Price*** | | | | | | | | | | | |  |
| PRI1 | The current price of energy is high compared to the average income | | | 1 | 2 | | 3 | | 4 | | 5 |  |
| PRI2 | The price of energy (electricity, gas) increases every year | | | 1 | 2 | | 3 | | 4 | | 5 |  |
| PRI3 | You feel worried when energy prices increase. | | | 1 | 2 | | 3 | | 4 | | 5 |  |
| ***VIII. Perceived of usefulness*** | | | | | | | | | | | |  |
| PU1 | Using appliances with energy-saving technology will help save on monthly electricity bills. | | | 1 | 2 | | 3 | | 4 | | 5 |  |
| PU2 | Using appliances with energy-saving technology will help protect the environment | | | 1 | 2 | | 3 | | 4 | | 5 |  |
| ***IX. Behavior control*** | | | | | | | | | | | |  |
| CON1 | You have the knowledge and skills to implement energy saving in your daily life. | | | 1 | 2 | | 3 | | 4 | | 5 |  |
| CON2 | Energy saving actions are easy for you. | | | 1 | 2 | | 3 | | 4 | | 5 |  |
| CON3 | Saving money is an important factor for you to implement energy-saving behaviors | | | 1 | 2 | | 3 | | 4 | | 5 |  |
| ***X. Attitude*** | | | | | | | | | | | |  |
| ATT1 | You think that saving energy in daily life will be helpful for environmental protection. | | | 1 | 2 | | 3 | | 4 | | 5 |  |
| ATT2 | You think that saving energy in daily life will help reduce greenhouse gas emissions. | | | 1 | 2 | | 3 | | 4 | | 5 |  |
| ﻿ATT3 | You think that saving energy in daily life is valuable to alleviate the current energy shortage problems. | | | 1 | 2 | | 3 | | 4 | | 5 |  |
| ***XI. Behavior*** | | | | | | | | | | | |  |
| BE1 | You turn off the device to reduce energy consumption when not in use | | | 1 | 2 | | 3 | | 4 | | 5 |  |
| BE2 | You used energy -saving appliances in your home. | | | 1 | 2 | | 3 | | 4 | | 5 |  |
| BE3 | You often remind others to use energy saving and efficiency | | | 1 | 2 | | 3 | | 4 | | 5 |  |
| **Part 2: Personal Information**  Please provide information by ticking the corresponding box below: | | | | | | | | | | | | |
| 1. Gender   ❒ Male | | | ❒ Female | | | | | | | | | |
| 1. Education   ❒ High School ❒ Colleage ❒ Graduate University ❒ Master/PhD | | | | | | | | | | | | |
| 1. Occupation   ❒ Offical staff ❒ Unemployment ❒ Worker ❒ Engineer  ❒ Self-employed        ❒ Lecturer/teacher. ❒ Others | | | | | | | | | | | | |
| 1. Income   ❒ under 10 million VND/month ❒ 10- under 15 million VND/month  ❒ 15- under 20 million VND/month. ❒ >20million VND/month | | | | | | | | | | | | |
|  | | | | | | | | | | | | |

**THANK YOU SO MUCH!**
